# Challenges and outcomes of upper cervical spinal tuberculosis surgery in pandemic–Case series study

**DOI:** 10.1016/j.ijscr.2024.109858

**Published:** 2024-06-06

**Authors:** Aries Rahman Hakim, Aries Rakhmat Hidayat

**Affiliations:** Department of Orthopedics and Traumatology, Faculty of Medicine, Universitas Airlangga/Dr. Soetomo General Academic Hospital, Surabaya, Indonesia

**Keywords:** Spinal tuberculosis, Anterior cervical corpectomy fusion, Occipito-cervical fusion

## Abstract

**Introduction and importance:**

Tuberculosis is a bacterial infection caused by *Mycobacterium tuberculosis*, primarily affecting the lungs. Conversely, the incidence of spinal tuberculosis (TB) was limited to a mere 6 % of cases of extrapulmonary tuberculosis. Upper cervical spinal TB is an exceptionally uncommon condition, with an incidence rate of approximately 0.3–1 % among all cases of spinal tuberculosis.

**Case description:**

Three patients diagnosed with upper cervical spinal tuberculosis who underwent Anterior Cervical Corpectomy Fusion (ACCF) or Occipitocervical fusion surgery were reviewed retrospectively. The data was obtained during the pandemic period in Indonesia. The patients were evaluated using pre-operative and post-operative Cobb's angles, Visual Analog Scale (VAS), Frankel scale, and Neck Pain and Disability (NPAD) scale.

**Clinical discussion:**

The ACCF surgery was more favourable when the compression was extended to the vertebral body; it showed good clinical and radiological outcomes. Multilevel ACCF and pathologies affecting bone quality seemed to be risk factors for material subsidence and instability. In this case, all the patients had performed ACCF surgery. The mean Cobb's angle pre-operative was 15.30, and Cobb's angle post-operative was 6.50. The mean pre-operative VAS value was 6.3, and the post-operative VAS value was 3. Compared to the post-operative scale, the pre-operative Frankel scale experienced an average increase of 2 levels. In contrast, the mean value of good post-operative NPAD is 29.3.

**Conclusion:**

Operative procedures on upper cervical spinal tuberculosis cases can improve patient's quality of life significantly, clinically and radiologically.

## Highlights

• The ACCF surgery showed good clinical and radiological outcome.
• The ACCF and ADCF are significantly favoured.
• The ACCF procedure has ability to produce adequate decompression.

## 1 Introduction and importance

Spinal TB is a commonly observed variant of the illness that occurs outside the lungs. Spinal tuberculosis is predominantly observed in immigrants from countries where the disease is widespread, particularly in affluent nations [1]. Although it could be in any segment of the spine, tuberculosis of the cervical spine is rare, comprising 3–5 % of cases of tuberculosis of the spine [2]. With adequate antibiotic treatment, cervical spine tuberculosis rarely causes mortality, but it commonly results in high rates of morbidity. [3]

There are several approaches to treating cervical spine tuberculosis, including the anterior approach, which is the most appropriate surgical approach for such cases, especially for patients in the early phase of bone destruction with or without kyphosis [4]. The Anterior Cervical Corpectomy Fusion (ACCF) is one of the treatment options for several cervical pathologies that, according to the studies, was a safe surgical treatment and has excellent outcomes for degenerative cervical pathologies [5,6]. In this paper, we report case series of the cervical spine during the pandemic, which was treated using ACCF. We constructed this case series using PROCESS checklist guideline [7].

## 2 Case presentation

This is retrospective review that includes three male patients diagnosed with upper cervical spinal tuberculosis (two patients with tetraparesis and one patient with tetraplegia). They underwent surgery, either anterior cervical corpectomy and fusion or occipitocervical fusion. The patients were reviewed retrospectively during the pandemic period from March 2021 to August 2022, and those who were followed up for at least three years were included in this study. All the procedure was done by the orthopedic consultant in our Hospital during pandemic period. We have obtained informed consent from each patient. The patients were evaluated using pre-operative and post-operative Cobb's angles, grading pain using the VAS, Frankel, and NPAD scales. All of our patients were diagnosed with spinal tuberculosis through a series of physical examinations, supplementary tests, and histopathological examinations. COVID-19 diagnosis has also been excluded through PCR test. All patients had received TB treatment for at least 9 months before undergoing surgery as an umbrella therapy based on Tuberculosis Guideline.

Table 1 shows the patient's demographics. The mean age was 28 years old (19–39). The mean Cobb's angle pre-operative was 15.30, and Cobb's angle post-operative was 6.50. The mean pre-operative VAS value was 6.3, and the post-operative VAS value was 3. Compared to the post-operative scale, the pre-operative Frankel scale experienced an average increase of 2 levels. Meanwhile, the mean value of good post-operative NPAD is 29.3. There was no complication recorded in our cases.

**Table 1:** Patient's demographic.

Case/sex/age (years old)	Cobb's angle pre-op	Cobb's angle post-op	VAS pre-op	VAS post-op	Frankel pre-op	Frankel post-op	NPAD pre-op	NPAD post-op
1/Male/19	0	3.6	6	2	C	E	23	2
2/Male/39	3.3	8.6	6	2	D	E	58	29
3/Male/26	42.6	7.5	7	2	A	E	80	57
**Mean value**	**15.3**	**6.5**	**6.3**	**3**	**–**	**–**	**53.66**	**29.3**

VAS = Visual Analogue Scale, NPAD = neck pain and disability scale.

### 2.1 Case 1

**Fig. 1:**
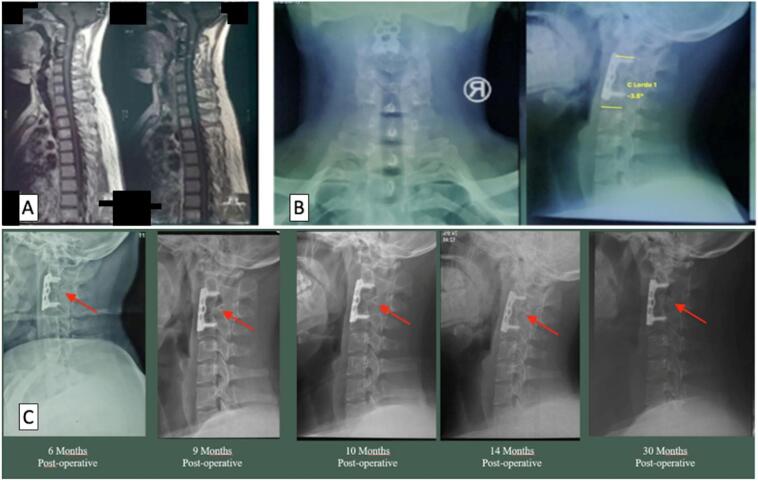
(A) Pre-operative cervical MRI; (B) Post-operative anteroposterior and lateral view cervical X-ray using ACCF on vertebrae cervical 3 with autologous iliac bone graft (red arrow); (C) Consecutive lateral cervical X-ray from 6 months post-operative to 30 months post-operative. Cervical fusion is observable in the last 6 months post-operative. Show fusion process on vertebrae cervical 3 with the implant remained intact and no failure was observed (red arrow). (For interpretation of the references to color in this figure legend, the reader is referred to the web version of this article.)

The first case was a 19-year-old male with chief complaint of neck pain and weakness on both hands and feet for six months before being admitted to the hospital. The cervical MRI (Fig. 1A) shows that the lesion was located on C2-C4. The patient diagnosed with tetraparesis Upper Motor Neuron (UMN) type due to upper cervical spine tuberculosis on vertebrae cervical 2,3,4 Frankel C. We attempted debridement and ACCF on vertebrae cervical 3 with autologous iliac bone graft augmentation. After the procedure, we found lordotic angles of 3.6° (Fig. 1B), VAS decreased to 2, Frankel scale improved from C to E, and NPAD score 2. A consecutive lateral cervical X-ray is displayed in Fig. 1C. Cervical fusion had been observable for six months post-operative.

### 2.2 Case 2

**Fig. 2:**
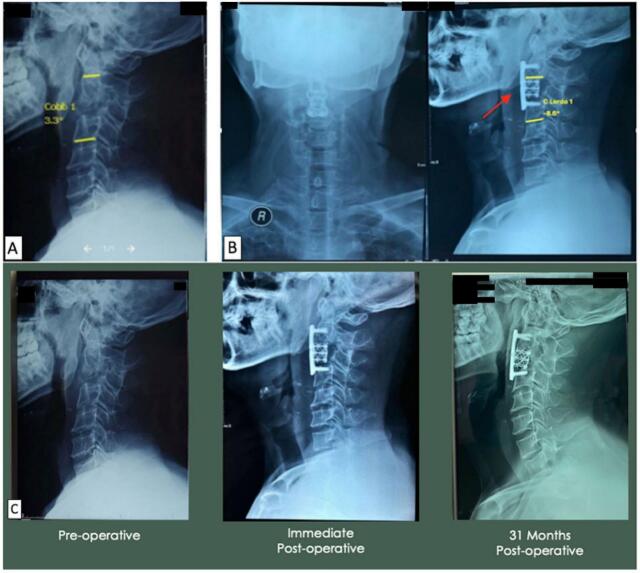
(A) Pre-operative lateral cervical X-ray; (B) Post-operative anteroposterior and lateral cervical X-ray using ACCF with mesh cage device (red arrow); (C) Consecutive lateral cervical X-ray from immediate post-operative to 31 months post-operative. Cervical fusion is observable, the fusion process on vertebrae cervical 3 using ACCF with mesh cage device remained intact and no failure was observed (red arrow). (For interpretation of the references to color in this figure legend, the reader is referred to the web version of this article.)

The second case was a 39-year-old male with chief complaint of neck pain for six months before hospital admission, patient felt numbness at the level of shoulders with weakness on both hands and feet. The pre-operative X-ray showed a 3.3° Cobb angle (Fig. 2A). The patient diagnosed with tetraparesis UMN type due to upper cervical spine tuberculosis vertebrae cervical 3 Frankel D. We attempted to do debridement and ACCF vertebrae cervical 3 with a mesh cage device. After the procedure, we found improvement of the deformity kyphotic 3.3° to lordotic 8.6° (Fig. 2B), VAS decreased to 2, improvement of Frankel scale to E, and NPAD score 29. A consecutive lateral cervical X-ray is displayed in Fig. 2C. Cervical fusion is observable, showing a fusion process on vertebrae cervical 3.

### 2.3 Case 3

**Fig. 3:**
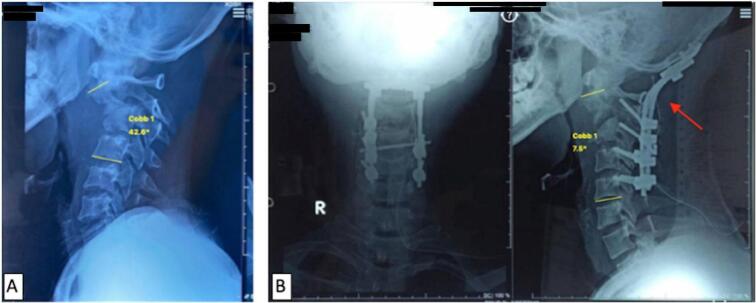
(A) Pre-operative lateral cervical X-ray; (B) Immediate postoperative anteroposterior and lateral cervical X-ray using posterior stabilization with occipital-cervical fusion (red arrow). (For interpretation of the references to color in this figure legend, the reader is referred to the web version of this article.)

**Fig. 4:**
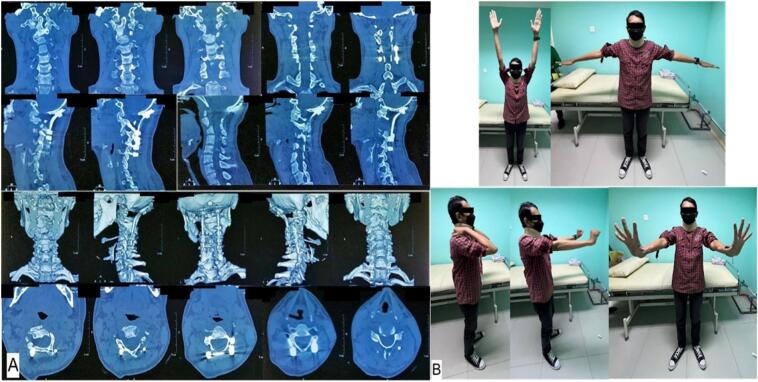
(A) Post-operative cervical CT-scan; (B) Post-operative range of motion shows improvement Frankel scale was observed 4 months post-operative.

**Fig. 5:**
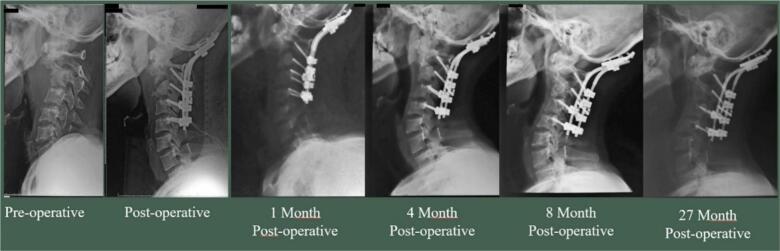
Consecutive X-rays from pre-operative to 27 months post-operative. Shows occipital-cervical fusion with the implant remain intact and was in proper position (red arrow). (For interpretation of the references to color in this figure legend, the reader is referred to the web version of this article.)

The last case was a 26-year-old male with chief complaint were neck pain for one year ago and weakness on both hands and feet for two months before admitted to the hospital. The X-ray shows a Cobb angle of 42.6° (Fig. 3A). The patient diagnosed with tetraplegic UMN type due to upper cervical spine tuberculosis on vertebrae cervical 1 – vertebrae cervical 4 with Frankel A. We attempted to do surgical decompression and posterior stabilization with occipitocervical fusion. After surgery, we found improvement in deformity kyphotic from 42.6° to kyphotic 7.5° (Fig. 3B), and VAS decreased to 2. Post-operative CT-scan evaluation showed the realignment and fusion using a lateral mass screw (Fig. 4A). Some remarkable improvement from the Frankel scale A to D and NPAD score from 80 to 57 (Fig. 4B). On long-term follow-up, occipital-cervical fusion with the implant remained intact and was in the proper position until 27 months post-operative (Fig. 5).

## 3 Discussion

The predominant skeletal expression of TB outside the lungs is most frequently observed in the spine, particularly in the thoracic and lumbar regions of the vertebral column. Instability and neurological impairments can arise in approximately 2–3 % of instances when the cervical spine is damaged, leading to lesions [[8], [9], [10]].

The best treatment for spinal tuberculosis is still debated and must be adjusted to the patient needs and condition [11]. Treatment includes antituberculosis medication and prevention of cervical collapse by nonsurgical or surgical means. Nonsurgical treatment with orthosis is the preferred treatment if there is no deformity or neurological impairment [12]. Surgery is indicated for diagnostic biopsy, abscess drainage, neural decompression, neurologic deficit, deformity correction, and stabilization of the affected spine [13]. The gold standard surgical treatment for cervical tuberculosis is decompression and anterior spine instrumentation [12,14]. Autograft augmentation can also be done to enhance bone healing speed, accelerate stability, and prevent deformity [11,12].

Anterior cervical corpectomy and fusion (ACCF) and anterior cervical discectomy and fusion (ACDF) are two successful surgical techniques used to alleviate pressure on the spinal cord in patients with severe narrowing of the spinal canal and to restore the natural curvature of the neck [5]. Anterior Cervical Discectomy and Fusion is superior to Anterior Cervical Corpectomy and Fusion when compression is limited to the disc level due to its advantages of reduced blood loss, shorter hospitalization, and fewer post-operative problems. Nevertheless, when the compression is expanded to the levels of the vertebral body, ACCF is significantly favoured over ACDF due to its ability to produce adequate decompression at the vertebral body levels [15,16].

In this case series, we assess the condition of the patient pre-operatively and then use ACCF and occipital-cervical fusion approach in order to debride and stabilize the cervical spine. All of the patients had remarkable improvement in the aspect of lordotic/kyphotic angle, pain and disability score (VAS and NPAD), and neurological function (Frankel). This is in conjunction with cohort studies in 10 patients with cervical spine tuberculosis who had been treated with the same methods. The surgical procedure demonstrated favourable clinical and radiological results, including successful bone fusion, proper alignment and preservation of the natural curvature of the spine, and the absence of any problems related to the implanted devices. This study shows that surgical intervention for decompression and stabilization of TB spondylitis in the cervical spine is a highly effective approach, yielding favourable neurological and radiological results [11].

Another study involving 119 patients with degenerative cervical pathologies who had been treated with ACCF showed that this procedure has low complication and revision rates. We can conclude that this procedure is a safe and effective treatment for degenerative and traumatic cervical spine disorders. Single-level ACCF can be carried out without the need for further posterior fusion (PF). Both multilevel ACCFs with more than two levels, as well as diseases that impair the quality of bone, appear to increase the risk of material sinking and instability [17]. In line with the ACCF technique used in 3 patients, the *occipital-cervical fusion* technique also showed a great result and improvement [18].

Two meta-analyses have shown that both ACDF and ACCF have positive results in terms of clinical measures, including the length of hospital stay, as well as scores on the Japanese Orthopedic Association (JOA) and Neck Disability Index (NDI) for multilevel Cervical Spondylitis Myelopathy (mCSM). The ACDF, due to its higher number of fixation points, provides a stronger level of rigidity to the construct than ACCF, which only has two fixation points. The ACDF exhibits improved radiographic outcomes at the last follow-up, including Cobb angles of C2 to C7, fusion rate, and graft subsidence [19].

In a previous retrospective analysis involving 140 patients, Audat et al. found no significant differences in clinical and radiological outcomes between the anterior and posterior surgical methods used to treat spondylotic cervical myelopathy. Nevertheless, the statistically significant disparities reported in the NDI for the anterior approach were not of clinical significance [19]. In contrast, a meta-analysis conducted by Zhu et al. found that the anterior method was more effective than the posterior approach in improving post-operative neural function for treating mCSM. While there was no apparent disparity in the rate of brain function recovery, the anterior group demonstrated considerably greater rates of complications and reoperations compared to the posterior group. Moreover, the surgical trauma related to corpectomy was much higher compared to that associated with laminoplasty/laminectomy. Therefore, it may be inferred that the anterior approach is more favourable than the posterior method for the treatment of CSM [20]. This case series has been reported in accordance with the PROCESS 2023 Criteria [7].

## 4 Conclusion

Operative procedures such as Anterior Cervical Corpectomy and Fusion (ACCF) on upper cervical spinal tuberculosis cases can improve patient's quality of life and give significant improvement clinically and radiologically. The improvement was shown on the lordotic/kyphotic angle pre-operative and post-operatively, the VAS score also improves, with change in the Frankel value and NPAD value.

## Informed consent

Appropriate consent was obtained from all individual participants included in the study.

## Funding

None.

## Author contribution

Aries Rahman Hakim involved in performing surgical technique, conceptualization, data curation, investigation, formal analysis, methodology, visualization, project administration, writing - original draft.

Aries Rakhmat Hidayat involved in performing surgical technique, conceptualization, data curation, investigation, formal analysis, methodology, visualization, project administration, writing - review and editing.

## Guarantor

Aries Rakhmat Hidayat

## Declaration of competing interest

The authors have no conflicts of interest to disclose.
